# Feline Conjunctival Sequestra—A Case Series

**DOI:** 10.1111/vop.70056

**Published:** 2025-07-27

**Authors:** Carolina Cauduro da Rosa, Brett D. Story, Sangwan Park, Gillian C. Shaw, Leandro B. C. Teixeira, Jacob Morris, Bret A. Moore, Fabiano Montiani‐Ferreira

**Affiliations:** ^1^ Comparative Ophthalmology Laboratory Federal University of Paraná, UFPR Curitiba Brazil; ^2^ Department of Surgical and Radiological Sciences, School of Veterinary Medicine University of California–Davis Davis California USA; ^3^ Comparative Ocular Pathology Laboratory of Wisconsin, School of Veterinary Medicine University of Wisconsin‐Madison Madison Wisconsin USA; ^4^ Department of Small Animal Clinical Sciences, College of Veterinary Medicine University of Florida Gainesville Florida USA

**Keywords:** cat, conjunctiva, cornea, necrosis, sequestrum

## Abstract

**Purpose:**

Corneal sequestrum is a condition commonly observed in cats, though the pathogenesis is not fully understood. The occurrence of conjunctival sequestrum has not previously been documented. This report aims to describe a series of cases of conjunctival sequestra in cats.

**Animals and Animal Samples Studied:**

A 3‐year‐old male Persian cat presented after 2 months of blepharospasm and epiphora in the right eye, accompanied by mild conjunctival hyperemia. Histopathologic findings from four other cases are also included.

**Methods:**

After presentation of Patient 1, the database (from 2007 to 2025) of the Comparative Ocular Pathology Laboratory of Wisconsin (COPLOW) was searched for other cases of feline conjunctival sequestrum, four of which were identified. In all cases, an amorphous black plaque was observed in the conjunctival tissue during the ophthalmological examination. Three of the four cases also had corneal sequestra ipsilaterally. Histopathology of the corneal sequestra was available in two of the cases.

**Results:**

Histopathology of all five conjunctival lesions revealed focally extensive compaction and brunescence of proprial collagen and absence of fibroblasts. Three of the cases had conjunctival ulceration overlying the sequestrum. The surrounding substantia propria was variably infiltrated by inflammatory cells and microorganisms. The appearance of conjunctival sequestra was remarkably similar to that of corneal sequestra.

**Conclusion and Clinical Relevance:**

This is the first reported case‐series of feline conjunctival sequestrum. Variable presentation of concurrent ocular abnormalities and history led to no definitive conclusion to a common cause. Similar to corneal sequestra, chronic irritation and a possible contribution from FHV‐1 infection are suspected.

## 1 Introduction

Corneal sequestrum (also known as corneal nigrum, corneal necrosis and corneal mummification) is a condition primarily observed in cats that is characterized by collagen degeneration and the accumulation of a distinctive brown pigment [1]. Brachycephalic breeds are predisposed to this disorder [2]. Clinical signs commonly include ocular discharge, blepharospasm, corneal ulceration, brown‐to‐black discoloration of the corneal stroma, vascularization, and inflammation [3]. Surgical intervention is typically the preferred treatment for corneal sequestra, aimed at alleviating patient discomfort and preventing further complications that may include a worsening stromal defect due to infection [4].

Corneal lesions are variable, but are most commonly described as discolorations ranging from transparent amber to opaque black, with or without corneal stromal vascularization [5]. Feline corneal sequestra mainly occur unilaterally; however, they may occur bilaterally, either simultaneously or at different times [5]. The pathogenesis of this disease is still unclear, but proposed etiologies include hereditary defect (Persian and Himalayan), Feline herpesvirus type 1—FHV‐1, and chronic irritation (e.g., adnexal disorders such as entropion, or tear film abnormalities) [5, 6, 7]. An association between FHV‐1 infection and the development of corneal sequestrum has been suggested but not proven; however, anatomical factors appear to play a more critical role in purebred brachycephalic cats [7, 8, 9].

The characteristic histopathologic finding of corneal sequestrum is coagulative necrosis of the corneal stroma with or without overlying ulceration of the corneal epithelium [8, 10]. Typically the anterior stroma is affected, though full‐thickness involvement can occur. The affected stroma lacks keratocytes, and its collagen fibers become compacted but retain their lamellar organization. Affected stroma resists enzymatic degradation. Blood vessels and inflammatory cells do not infiltrate sequestra, but bacterial or fungal colonization is common. At the periphery of a sequestrum, varying degrees of inflammatory cell infiltration, blood vessel in‐growth, edema, and granulation tissue formation are commonly observed [10].

The present study aims to describe five cases of conjunctival sequestrum in cats, highlighting this previously overlooked and rarely discussed clinical condition in veterinary ophthalmology. A case of palpebral conjunctival sequestrum combined with corneal sequestrum was diagnosed in a Persian cat presented to the Laboratory of Comparative Ophthalmology (LABOCO) at the Veterinary Hospital of the Federal University of Paraná (UFPR). The four additional cases—two affecting the palpebral conjunctiva and two involving the conjunctiva of the third eyelid—were identified in the archives of the Comparative Ocular Pathology Laboratory of Wisconsin (COPLOW, Madison, WI), based on a review spanning 2007 to 2025. This study complies with the Guidelines for Ethical Research in Veterinary Ophthalmology (GERVO) and is exempt from approval by an ethics committee. Animal owners or owners' representatives provided written consent for the treatment provided and for the publication of data and images. The authors declare that there are no conflicts of interest related to this work.

## 2 Clinical Case Report

### 2.1 Examination Findings and Surgical Treatment

A 3‐year‐old male Persian cat (Patient 1) was referred for a dark plaque on the right cornea. The cat had originally developed blepharospasm and epiphora in the same eye 2 months earlier, accompanied by mild conjunctival hyperemia. The ophthalmological examination was performed following a systematic sequence that consisted of a menace response test, pupillary light reflex, Schirmer Tear Test (Teste Lacrimal de Schirmer, Ophthalmos, SP, Brazil), slit lamp biomicroscopy (PLS Portable Slit Lamp, Keleer, Pennsylvania, USA), fluorescein staining test (1% sodium fluorescein, Oftalmopharma, SP, Brazil), intraocular pressure (Icare TonoVet, Finland) and indirect ophthalmoscopy with a 20D lens (Optical Lens V20LC, Volk, Ohio, USA) in both eyes.

**FIGURE 1:**
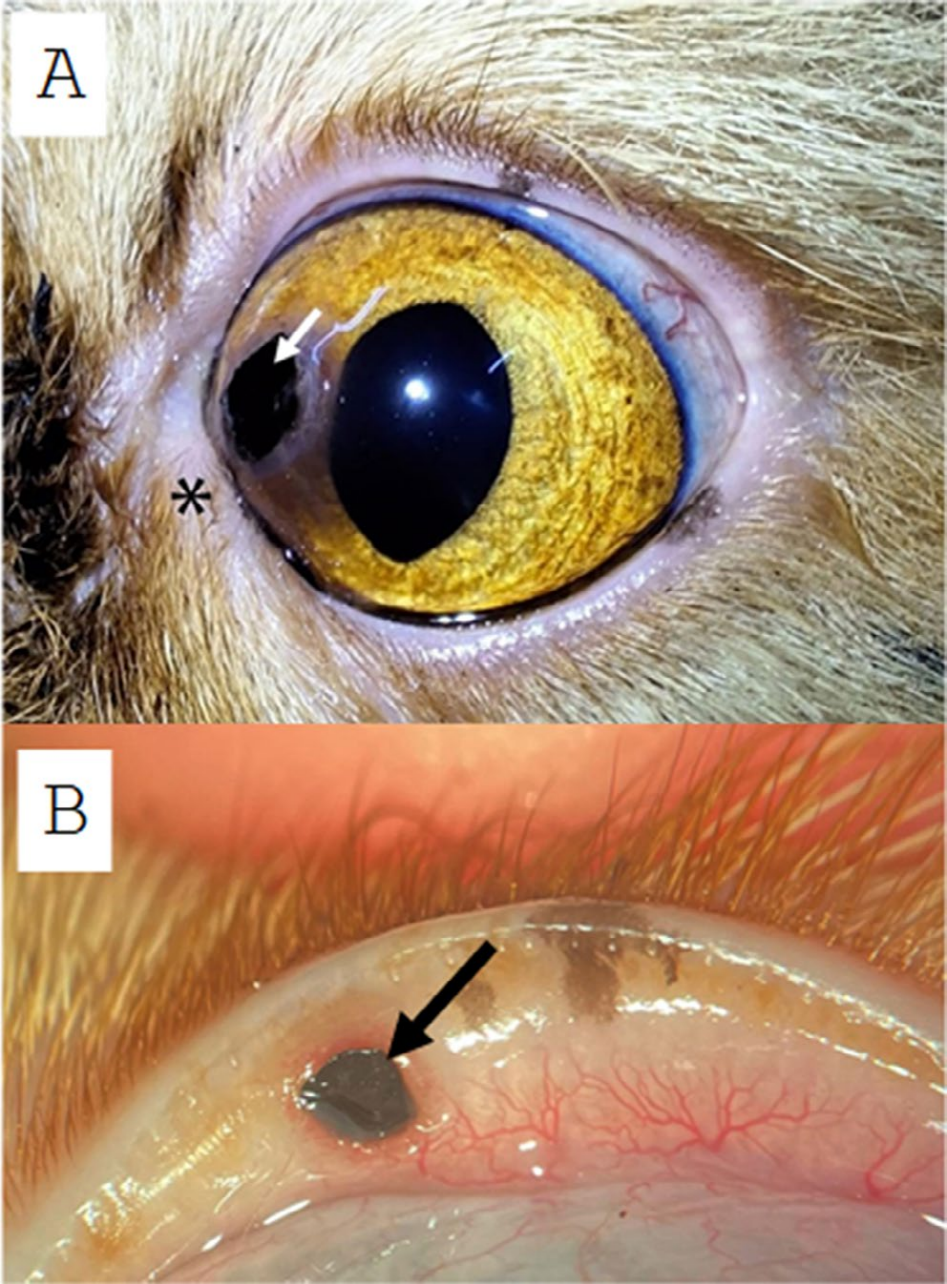
Right eye of a 3‐year‐old male Persian cat (Patient 1). (A) Corneal sequestrum presenting as a dark plaque in the medial cornea (white arrow—corneal sequestrum), with surrounding white corneal opacity and medial canthal entropion (asterisk). (B) Sequestrum on the palbebral conjunctiva of the everted right upper eyelid. Note the amorphous black plaque measuring approximately 3 mm in diameter (black arrow) and located approximately 2 mm from the eyelid margin. The conjunctival tissue was hyperemic around the lesion.

A dark lesion on the cornea with associated amber discoloration involving approximately 1/15th (approximately 7%) of the corneal surface was found on the right eye and was consistent with corneal sequestrum (Figure 1A). There was also mild medial canthal entropion of the same eye. Additionally, another amorphous black plaque was observed on the upper palpebral conjunctiva of the same eye, measuring approximately 3 mm in diameter (Figure 1B). Surgical removal of both the corneal sequestrum and conjunctival lesion was recommended. Until the date of the procedure, a lubricating eye drop containing sodium hyaluronate 0.15% was used (Hyabak, União Química, São Paulo, SP, Brazil). Slit lamp biomicroscopy revealed no other lesions of the anterior segment. Funduscopic examination revealed no lesions of the posterior segment. The remainder of the physical examination was unremarkable.

Patient 1 presented for surgery 2 weeks following the initial consultation. A surgical microscope (MC‐M2222; D.F. Vasconcelos, São Paulo, SP, Brazil) was used with the patient under general anesthesia. First, a superficial lamellar keratectomy was performed to remove the corneal sequestrum, followed by excisional biopsy of the dark plaque from the upper palpebral conjunctiva using 11 cm curved Castroviejo scissors. Due to the minimal depth (< 50%) of the corneal and conjunctival lesions, no additional graft techniques were performed. Both corneal and conjunctival plaques were preserved in 10% buffered formaldehyde to be submitted for histologic review. A medial canthoplasty combined with a modified Hotz‐Celsus procedure was performed to correct the right inferior medial entropion. Postoperative therapies included 0.3% tobramycin ophthalmic solution OD q6 hours and lubricating drops OD q4 hours.

Recheck evaluation 7 days postoperatively showed that both surgical sites were healing as expected for this time period. At 12 weeks postoperatively, there was no evidence of ocular pain in the cornea or the upper palpebral conjunctiva, there was no recurrence of sequestrum formation, and a marked improvement in corneal transparency was noted.

### 2.2 Histological Analysis

**FIGURE 2:**
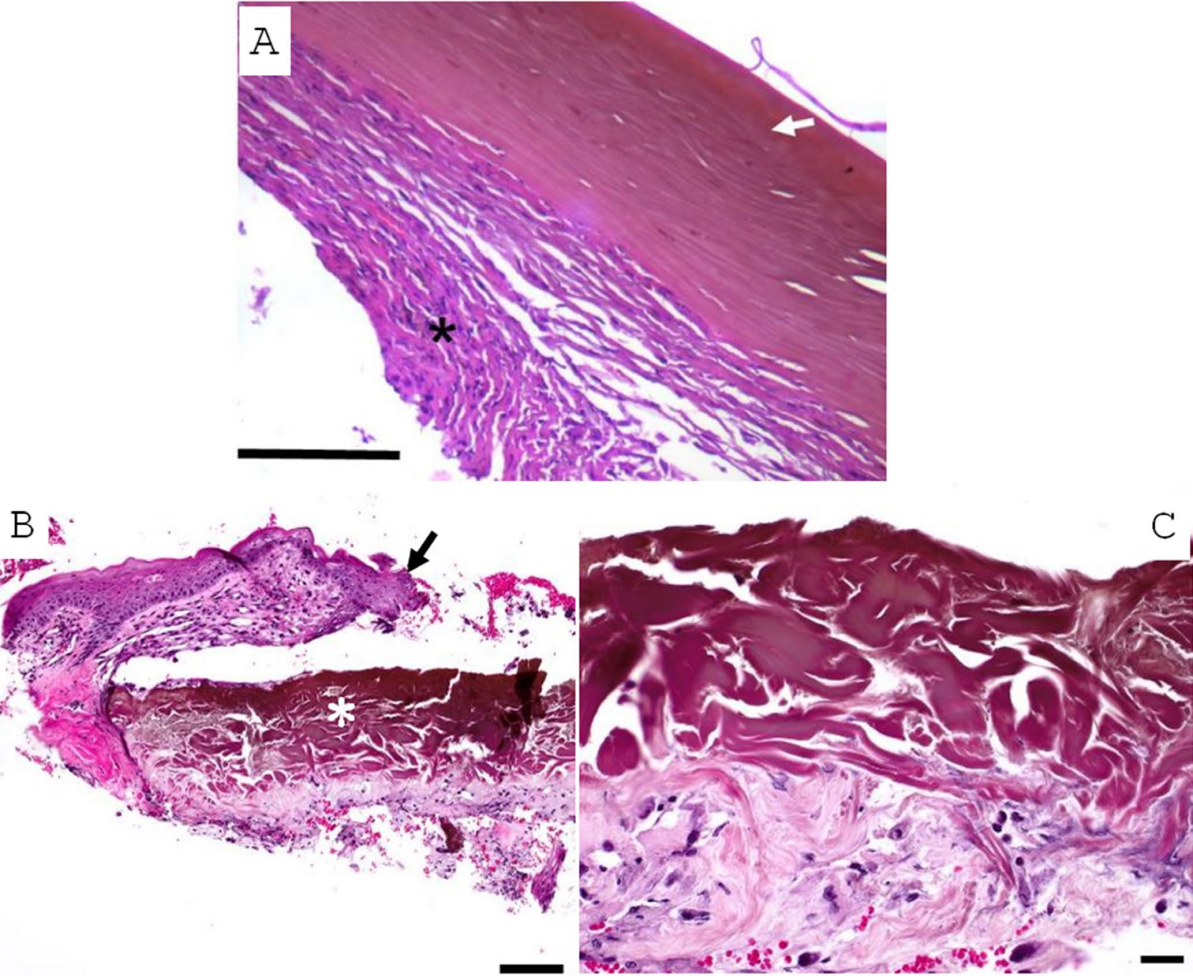
(A) Histologic section of the corneal plaque removed by superficial lamellar keratectomy in Patient 1. There is brownish discoloration and compaction of the collagen lamellae and lack of viable keratocytes in the affected area (arrow). Inflammatory cells infiltrate the surrounding stroma (asterisk). The surface (top of the image) lacks epithelium (ulceration). Hematoxylin and eosin stained. Scale bar 100 μm. (B, C) Histologic section of the dark conjunctival lesion removed by excisional biopsy in Patient 1. (B) The overlying conjunctival epithelium is discontinuous (ulceration) (black arrow) and separated from the sequestrum (indicated by white asterisk). Hematoxylin and eosin stained. Scale bar 100 μm. (C) There is brownish discoloration and compaction of the collagen and lack of viable fibroblasts in the sequestrum and an abrupt transition between affected and unaffected tissue. A small number of inflammatory cells and nuclear debris occupy the surrounding substantia propria. Hematoxylin and eosin stained. Scale bar 20 μm.

Both the corneal and conjunctival samples from Patient 1 were submitted to the Comparative Ocular Pathology Laboratory of Wisconsin (COPLOW) for review and routinely processed for histopathology. Morphologic features of the cornea included superficial ulceration, brownish discoloration of the anterior stroma, and compaction of lamellar collagen. The collagen lamellae were compacted. Neutrophils and blood vessels infiltrated and expanded the stroma around the sequestrum, which was also edematous at the periphery (Figure 2A). The dark conjunctival plaque consisted of compacted proprial collagen with brownish discoloration, absence of viable cells, and overlying ulceration (Figure 2B,C). The overlying conjunctival epithelium and substantia propria were discontinuous, indicating ulceration. Inflammatory cells including lymphocytes, plasma cells, and neutrophils, along with numerous blood vessels and plump spindle cells (fibroblasts) populated the surrounding substantia propria. These findings confirmed the diagnosis of a conjunctival sequestrum.

## 3 Additional Histological Cases

**FIGURE 3:**
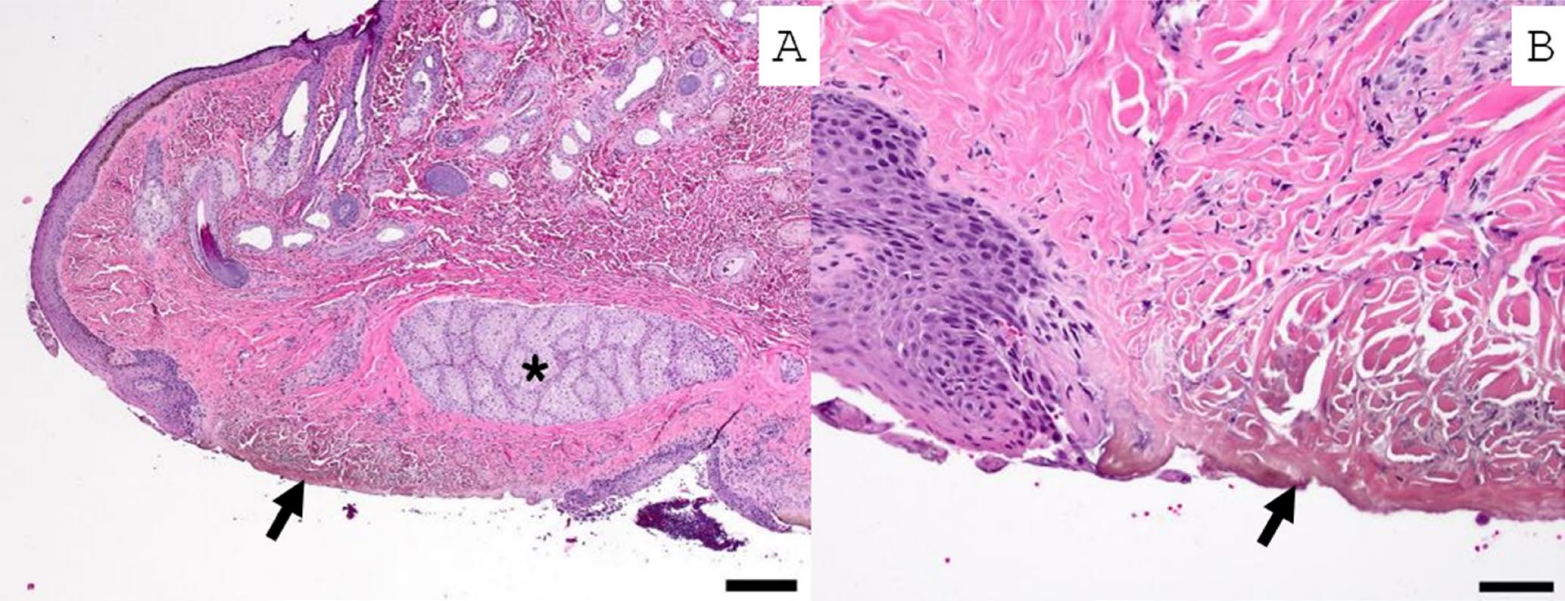
Sample from the lower eyelid conjunctiva, removed by excisional biopsy, from Patient 2 demonstrating a focal area of brown discoloration at the surface. (A) There is focally extensive conjunctival epithelial ulceration with compaction and brunescence of the underlying conjunctival proprial collagen (black arrows). Note the meibomian gland (asterisk). Hematoxylin and eosin stained. Scale bar 200 μm. (B) Higher magnification image of lesion. A few inflammatory cells infiltrate the surrounding substantia propria. Hematoxylin and eosin stained. Scale bar 50 μm.

Besides the case reported, four additional cases of feline conjunctival sequestrum were identified from samples submitted to the Comparative Ocular Pathology Laboratory of Wisconsin (COPLOW) (Table 1). Patient 2 was an 8‐year‐old female spayed domestic shorthair (DSH) originally evaluated at a private veterinary ophthalmology clinic. She presented with a corneal sequestrum, keratitis, and lower lid entropion, as well as a pigmented lesion on the lower palpebral conjunctiva of the left eye. Histologic findings of the conjunctival lesion included a focal area of conjunctival ulceration and underlying proprial collagen compaction and brunescence (Figure 3).

**TABLE 1:** Signalment, location, affected eye, and key clinical features of 5 conjunctival sequestra in cats.

Patient	Sex	Breed	Age (years)	Sequestrum location	Affected eye	Corneal sequestrum history	Other clinical features
1	M	Persian	3	Upper palpebral conjunctiva	Right	Ipsilateral eye	Blepharospasm, epiphora, medial canthal entropion
2^a^	FS	Domestic Shorthair	8	Lower palpebral conjunctiva	Right	Ipsilateral eye	Vascularized corneal lesion, lower lid entropion
3^a^	MN	Domestic Shorthair	6	Third eyelid conjunctiva	Right	Contralateral eye	Bilateral entropion, third eyelid hyperemia
4^a^	MN	Persian	1.7	Lower palpebral conjunctiva	Right	Ipsilateral eye (also prior in same eye)	Firm black subconjunctival mass, prior corneal sequestrum
5^a^	FS	Domestic Medium Hair	10	Third eyelid conjunctiva	Both	Absent	Black plaques on third eyelids; presumed chronic FHV‐1

^a^ From the archives of (xx removed for anonymity xx).

**FIGURE 4:**
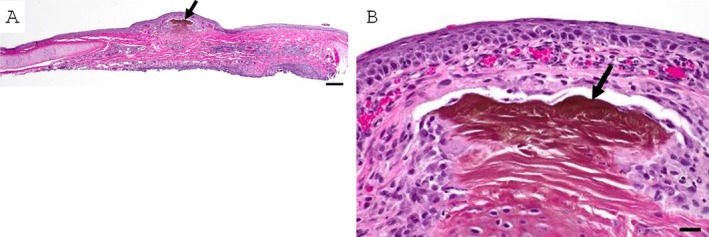
Third eyelid conjunctival sequestrum removed by excisional biopsy from Patient 3, at lower (A—scale bar 200 μm) and higher (B—Scale bar 20 μm) magnification. The affected area (black arrow) is compacted proprial collagen with brunescence. The surrounding substantia propria is hypercellular and heavily vascularized (granulation tissue). Another similar lesion is located out of the plane of section. The epithelium was intact in the examined sections.

**FIGURE 5:**
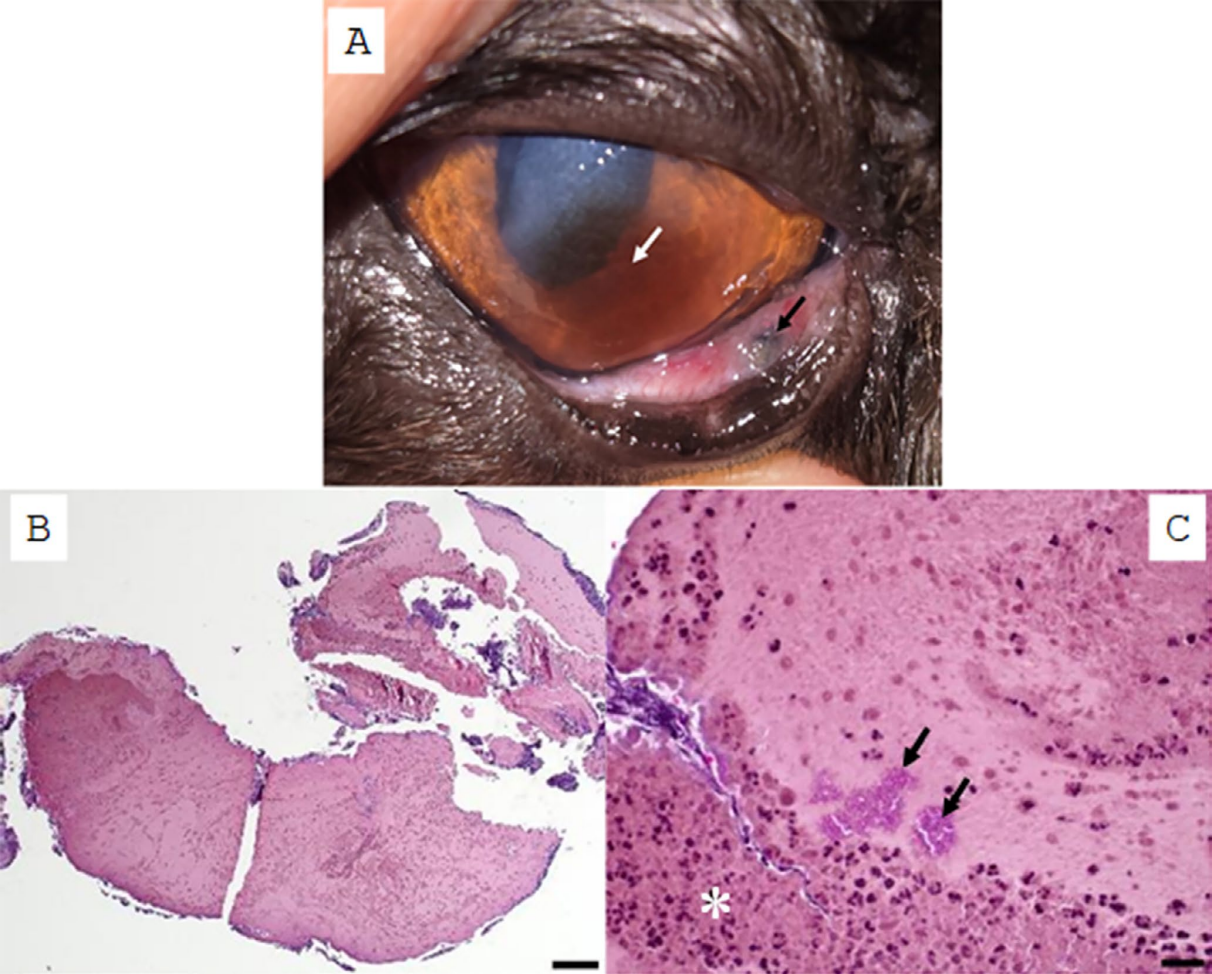
Corneal and conjunctival sequestra in the right eye of Patient 4. (A) Clinical image of the right eye with sequestra of the cornea (white arrow) and palpebral conjunctiva (black arrow). (B) Photomicrograph of the focal black discoloration on the lower eyelid conjunctiva. The proprial collagen is compacted, slightly brown and lacks fibroblasts. The conjunctival epithelium is absent (ulceration). Hematoxylin and eosin stained. Scale bar 200 μm. (C) Higher magnification image of lesion. Degenerate neutrophils (*) and a few bacterial colonies (black arrows) infiltrate the tissue. Hematoxylin and eosin stained. Scale bar 20 μm.

Patients 3 and 4 were originally evaluated at the Comparative Ophthalmology Service at the University of California Davis (UC Davis), School of Veterinary Medicine. Patient 3 was a 6‐year‐old neutered male DSH that was presented for evaluation of a corneal sequestrum of the left eye, presumed secondary to chronic bilateral entropion leading to non‐ulcerative keratitis in both eyes. Additionally, two pigmented foci with surrounding hyperemia on the conjunctiva at the leading edge of the third eyelid in the right eye were appreciated. Histologically, the conjunctival lesion was a focal area of conjunctival proprial collagen compaction and brunescence. The epithelium was intact overlying the sequestrum in the examined sections (Figure 4). Both eyes displayed macroblepharon, and the bilateral entropion was presumed secondary to acromegaly. Entropion was corrected by medial canthoplasty combined with a modified Hotz‐Celsus, following lamellar keratectomy and conjunctival excisional biopsy of the darkly pigmented lesions. Patient 4 was a 1.7‐year‐old neutered male Persian cat that was diagnosed with a ventronasal paraxial corneal sequestrum of the right eye. A corneal sequestrum had previously been diagnosed in the dorsotemporal paraxial cornea of the same eye but had spontaneously resolved with medical treatment 16 months prior to re‐presentation. Additionally, a small well‐circumscribed firm black lesion was appreciated under the lower palpebral conjunctiva near the medial canthus in the same eye (Figure 5). Excisional biopsy of the subconjunctival black mass revealed brown‐tinged eosinophilic acellular material with neutrophilic infiltration and bacterial colonization (Figure 5).

**FIGURE 6:**
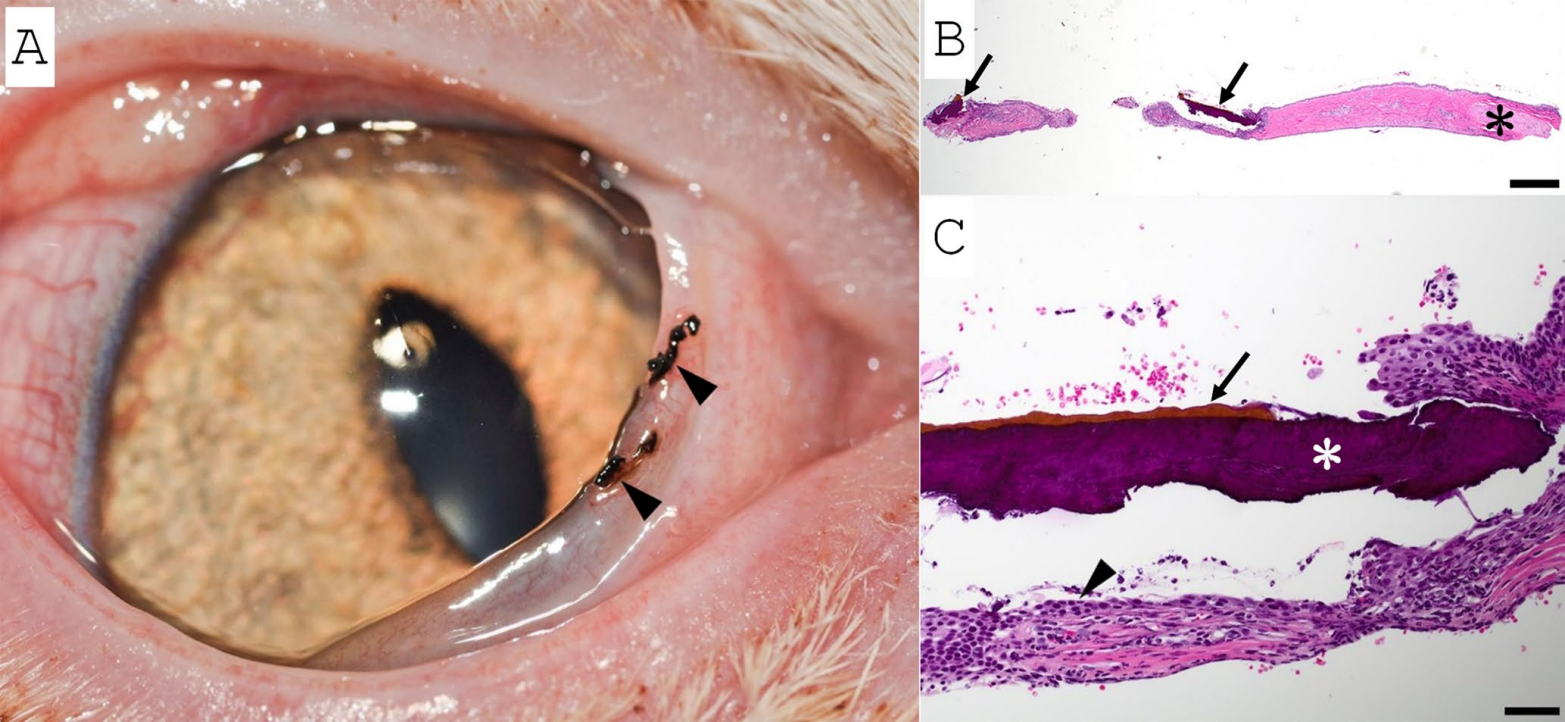
Conjunctival sequestrum in Patient 5. (A) Gross photographs showing dark plaques (black arrowheads) on the anterior surface of the right third eyelid toward the leading edge. (B) The black plaques noted clinically correspond histologically to areas of ulceration overlying mineralized conjunctival substantia propria with adherent brown material (black arrows). Black asterisk indicates the cartilage of the leading edge of the third eyelid. Scale bar 500 μm. Hematoxylin and eosin stained. (C) The surface of the mineralized substantia propria (white asterisk) has a thin layer of adherent brown material (black arrow). The conjunctival epithelium extends under the sequestrum (black arrowhead). Hematoxylin and eosin stained. Scale bar is 50 μm.

Patient 5 was a 10‐year‐old spayed female domestic medium‐haired cat evaluated at the University of Florida, with a history of presumed chronic herpesvirus. Brown plaques on the anterior surface of both third eyelids were evident (Figure 6), along with mild conjunctival hyperemia. The patient did not have other corneal, eyelid, or conjunctival abnormalities. The leading edge of the right third eyelid was surgically excised. Histopathological findings included conjunctival ulceration with underlying mineralization of the conjunctival substantia propria with an adherent layer of brown material and mild lymphoplasmacytic and neutrophilic conjunctivitis (Figure 6).

## 4 Discussion

Although conjunctival sequestration in cats, including the third eyelid and conjunctival grafts, has been anecdotally described by veterinary ocular pathologists [11], to the authors' knowledge, this is the first documented report of conjunctival sequestra in cats. The five cases presented here show a spectrum of presentations and concurrent ocular pathologies, most notably the presence of corneal sequestra in four of the eyes. We suggest that the pathogenesis of conjunctival sequestration may be similar to the still not fully understood pathogenesis of corneal sequestration.

Feline corneal sequestra are not uncommon, and have been identified in many different cat breeds. Brachycephaly has been speculated to increase susceptibility, although a genetic predisposition is also possible [12, 13, 14]. Conformational abnormalities that often occur with brachycephaly but can also occur in non‐brachycephalic breeds, such as entropion, may represent significant predisposing factors in the occurrence of corneal sequestra [2, 8]. Entropion was observed in three of the four cases presented here that had corneal sequestra. One report found no significant association with altered conjunctival goblet cell numbers, qualitative tear film abnormalities, or tear film break‐up time as contributory factors in the development of feline corneal sequestrum [15]. Although no causal relationship has yet been established, corneal sequestrum has historically been suspected to be associated with FHV‐1 [16]. Given that FHV‐1 infection can cause corneal and conjunctival ulceration, it may play a role in the formation of sequestra [17]. The infectious disease status of the five feline patients in the present study is unknown.

An alternative explanation for the cause of conjunctival sequestra is that corneal sequestra are detaching from the cornea and subsequently becoming embedded within the conjunctival tissue. This possibility cannot be entirely excluded, as spontaneous sloughing of corneal sequestra has been documented [8, 18]. However, we suggest this possibility is very unlikely, considering that in Patient 4 the corneal sequestrum was observed contralateral to the conjunctival sequestrum of the third eyelid, and in Patient 5 sequestra were found on the leading edges of both third eyelids without any keratitis or evidence of previous corneal sequestra. Patient 4 had bilateral entropion, which may have contributed to the formation of the conjunctival lesion.

In human patients, conjunctival necrosis itself is a rare complication of the long‐term use of topical epinephrine eye drops (first reported in 1927 [19]), topical antibiotics [20], and subconjunctival [21] or intravitreal corticosteroids [22, 23]. Microbial infections, chemical burns, and cryosurgery have also been incriminated [24]. No known histories of any of the above were evident in the cases presented here, although detailed historical information was not present for some of the cases and thus cannot be completely ruled out.

The basis of the dark discoloration of corneal sequestra remains debatable, with porphyrins, naturally present in feline tears, being cited as a possible cause [18]. Although melanin has been recently proposed as a contributing factor based on absorbance spectra and optical microscopy [25], ultrastructural analysis has found no evidence of melanosomes [11]. The similar brown discoloration of conjunctival lesions is presumed to develop in the same manner as that of corneal sequestra, but pathogenesis remains unknown. Overall, the histological findings of the conjunctival sequestra are very similar to those seen in corneal sequestra.

A presumptive diagnosis of corneal sequestra is generally made based on clinical examination as they have a characteristic appearance. However, in cases involving the conjunctiva, diagnostic tools such as surgical biopsy followed by histological analysis are essential for differentiating it from conditions like a melanocytic neoplasm. Given the considerable difference in prognosis, accurate diagnosis is crucial for appropriate management and treatment. No recurrence was observed in any of the conjunctival sequestrum cases reported here, indicating a favorable surgical outcome.

The recommended treatment of corneal sequestra depends on the size, depth, potential for self‐extrusion or resolution, and the level of discomfort experienced by the patient. If untreated, some sequestra can be extruded over time, leaving a stromal ulcer to be healed, some that can be very deep or even penetrating [9, 10, 14]. Corneal sequestra may be painful and may take several months to slough naturally, and in many cases, surgical removal accelerates healing and provides prompt relief [26]. It is possible that conjunctival sequestra may also be associated with discomfort. Excisional biopsy serves both as a diagnostic tool and a potentially curative treatment. In general, prognosis is more favorable with early diagnosis. The complete removal of the dark plaque and debridement of devitalized tissue should lead to complete healing with proper management [27]. There is some evidence suggesting that covering the keratectomy site with a graft or flap can provide structural support, promote healing, and potentially reduce the risk of recurrence. Techniques such as conjunctival grafts, amniotic membrane transplantation, and synthetic grafts are recommended based on the lesion's depth and location [5, 28, 29, 30]. The early stage and small size of the lesion of the 3‐year‐old male Persian patient discussed in this report required no debridement or sutures after surgical removal, which may have prevented some secondary complications or recurrence.

Due to the lack of understanding of the pathogenesis of corneal sequestration, it is possible to infer that other alterations associated with this condition may still be unknown or even underdiagnosed. This study suggests that the development of conjunctival sequestra may be similar to that of corneal sequestra, and recommends that further studies be conducted to clarify this association, as well as its impact on the ocular health of cats. The two cases in this series that had a conjunctival sequestrum without ipsilateral corneal sequestrum suggest that an associated corneal sequestrum is not required for the development of conjunctival sequestrum. Furthermore, the conjunctiva of feline patients with corneal sequestra and/or eyelid entropion should be carefully inspected for corresponding conjunctival lesions. Finally, any brown‐black discoloration of the feline conjunctiva should always undergo laboratory analysis to accurately differentiate melanosis from neoplasms and sequestrum (necrosis), as described herein, ensuring a precise diagnosis and appropriate management.

## Ethics Statement

This study complies with the Guidelines for Ethical Research in Veterinary Ophthalmology (GERVO) and is exempt from approval by an ethics committee.

## Consent

Animal owners or owners' representatives provided written consent for the treatment provided and for the publication of data and images.

## Conflicts of Interest

The authors declare no conflicts of interest.

## Data Availability

The data that support the findings of this study are available from the corresponding author upon reasonable request.
